# A 6-year retrospective clinical review of iatrogenic ureteric injuries repaired in a resource-deprived setting

**DOI:** 10.1186/s12893-022-01817-3

**Published:** 2022-11-05

**Authors:** Mahamudu Ayamba Ali, Raymond Saa-Eru Maalman, Mawuenyo Attawa Oyortey, Yaw Otchere Donkor, Kekeli Kodjo Adanu, John Tampuori, Mathew Yamoah Kyei

**Affiliations:** 1 Departments of Surgery and Basic Medical Science, School of Medicine, University of Health and Allied Sciences, Ho, Volta Region Ghana; 2 Ho Teaching Hospital, Ho, Volta Region Ghana; 3 Department of Surgery, University of Ghana Medical School, Accra, Ghana

**Keywords:** Ureteral injury, Iatrogenic ureteral damage, Ureteroneocystostomy (reimplantation), Hysterectomy

## Abstract

**Background:**

Seventy percent of ureteric injuries result from iatrogenic causes with about 75% of these diagnosed in the postoperative period. It may have fatal complications such as sepsis and or renal functional damage increasing morbidity and treatment cost.

**Objective:**

The study aimed to identify the risk factors for iatrogenic ureteric injuries from open surgical procedures and the intervention outcome in a resource-poor setting.

**Patients and methods:**

This was a multi-centre study. The clinical records of patients with iatrogenic ureteric injuries seen between 2015–2021 who were managed at the urology units of the Margaret Marquart Catholic Hospital, and the Ho Teaching Hospital, in the Volta region of Ghana, were retrieved. The data extracted included patients’ demographic factors, the clinical presentation, the primary surgery details, the time from surgery to presentation, the intervention offered, and the outcomes. The data were analysed using the Statistical Package for Social Scientists (SPSS) version 24.0.

**Results:**

Twelve patients aged between 24–54 years with a total of 19 ureteric injuries were managed. The injuries resulted from a hysterectomy in 10 cases (83.3%), and one each from emergency caesarean section and inguinal hernia repair with traction and transection injuries respectively (16.7%). Seven out of 12 cases were diagnosed 48 h after surgery. Bilateral injuries occurred in 7 cases (14/19 injuries). Intraoperative recognition was common in unilateral injuries and surgeries performed by specialist surgeons. Ureteroneocystostomy (14/19), uretero-ureterostomy (1/19), and open suture release were the management procedures performed as in the intervention.

**Conclusion:**

Open hysterectomy (83.7%) was the most common procedure leading to iatrogenic ureteric injuries in this study. Intra-operative recognition occurred when trained specialist surgeons performed the surgery. Late presentation with more severe morbidity was found amongst non-specialist surgeons. Thus, improvement in training to allow intra-operative diagnosis should be encouraged in general practitioners to reduce morbidity and improve outcomes.

## Background

Iatrogenic ureteral injury (IUI) is a potentially devastating complication of both open and endourological surgery. It remains largely unnoticed particularly in surgeries in the setting of recurrent pelvic operations, pelvic inflammatory diseases, and tumours [1]. The incidence of IUIs is reported to occur in 1.3 per 1000 gynaecologic surgery [2–4]. Intraoperative diagnosis often poses a challenge as intraoperative haemorrhage, adhesions and abnormal course of ureters may obscure injury signs or prevent direct ureteral exploration leading to over 70% of the cases diagnosed after surgery [5, 6]. In addition, the lack of endourological set-ups in most resource-deprived facilities for standard intraoperative ureteric delineation after major pelvic surgery makes damage diagnosis difficult [7]. Delayed diagnosis of the injury precludes immediate repair and predisposes the patient to intra-abdominal sepsis, flank pains, kidney damage, and ureteral stricture formation with potential medical litigation [6, 8]. The limited kidney replacement therapy and ureteral re-constructive centres, the high treatment cost especially in low-income areas may further predispose these patients to mortality from complications. To reduce these complications and subsequent morbidity associated with IUIs, knowledge of preventive factors or factors that enhance early recognition is very important. Analysing these injuries will give clinicians an understanding of factors that lead to unintended ureteric injury and those that influenced delayed diagnosis.

We present a retrospective study evaluating 6 years of IUI from open surgery requiring surgical repair. Open pelvic surgery is performed frequently in low-resource centres where laparoscopic services are often lacking and is a definitive way of diagnosing, classifying, and repairing most of these injuries. We believe the findings in this study will help shape practice in identifying and managing ureteral injuries after open surgeries.

## Methodology

### Study design

This was a multi-centre retrospective study involving the retrieval and analysis of medical records of IUI patients who were managed at the Margaret Marquart Catholic Hospital (MMCH), Kpando, and Ho Teaching Hospital (HTH), Ho in the Volta Region of Ghana between January 2016 to October 2021. These two hospitals had urologists who received and managed IUIs from most parts of the Volta Region of Ghana.

Patient’s records that were included in the study satisfied the following criteria:

1. The patient had open surgery at a primary facility.
2. The ureteral injury was confirmed and had been repaired.
3. Complete records of biodata information, indications for the first surgery, diagnosis of IUI, repair, and complications.

Ureteral repairs for patients with congenital anomalies, other trauma, and patients with inadequate information were excluded. The study was approved by the Ho Teaching Hospital Research and Ethical Committee with reference No. HTH/RPPME/E-1 and approval identity HTHREC (15) FC-2022. Verbal and written informed consent was obtained from all patients in both institutions for the extraction of data and use for publication.

### Data extraction

The data extracted from patients’ folders included age, sex, primary surgery records and any pre-operative imaging of ureters, clinical presentation, resuscitation required, types of injuries, evidence of previous adhesions separated (previous surgical history, raw areas, fibrous tissues at points of dissection recorded), multiple suturing at vascular areas for intraoperative bleeding, postoperative blood loss, wound infection, re-laparotomy, renal function, deep vein thrombus formation, days spent in the hospital, and any mortality. The source of information included the emergency registers, theatre, and admission records. The data was analysed using SPSS version 24.0 and presented as descriptive statistics using frequencies and percentages.

## Results

### Characteristics of cases under review

A total of 12 cases were identified with a mean age of 37.5 ± 9.3 years (range 24 to 59 years). Twenty-five percent (21% injuries) of the cases were within the age of 20–29 years while 41.7% (42.2% injuries) were between 30–39 years. Almost all injuries (94.7%) occurred in females (11/12). Elective abdominal hysterectomy for uterine fibroids was the main indication for primary surgery found in 8 (66.7%) cases. Out of 919 hernia repairs done one emergency inguinal hernia repair (0.1%) resulted in ureteric injury at the centre where the injury occurred (Table 1). None of the patients had ureteric imaging or delineation before primary surgery.

**Table 1:** Characteristics of cases reviewed

Variables	Case frequency (N = 12)	Injury freq. (%) (total = 19)
Age		
20–29	3 (25.0)	4 (21.0)
30–39	5 (41.7)	8 (42.2)
40–49	2 (16.6)	3 (15.8)
50–59	2 (16.6)	4 (21.0)
Sex		
Male	1 (8.3)	1 (5.3)
Female	11 (91.7)	18 (94.7)
Primary diagnosis		
Fibroid (ultrasound scan diagnosis)	8 (66.7)	13 (68.4)
Abnormal uterine bleeding	1 (8.3)	2 (10.5)
Postpartum haemorrhage	1 (8.3)	2 (10.5)
Obstructed labour (solitary kidney)	1 (8.3)	1 (5.3)
Right obstructed inguinal hernia	1 (8.3)	1 (5.3)
Primary surgery that resulted in the ureteric injury		
Hysterectomy (abdominal)	10 (83.3)	17 (89.4)
Caesarean section	1 (8.3)	1 (5.3)
Herniorrhaphy	1 (8.3)	1 (5.3)

With regards to the level of training of the persons that performed the primary procedures, the majority (58.3%) of the primary surgeries that resulted in IUI were performed by general physicians (MBChB and equivalent certification who were medical officers) with only a quarter (25%) of the cases being performed by a specialist (either an obstetric gynaecologist or general surgery specialists surgeon specialist). Two out of the 12 cases (16.7%) were performed by a public health specialist. Ninety-two percent of the cases performed by medical officers resulted in bilateral IUIs. Two out of the three IUIs (66.7%) caused by public health specialists were bilateral IUIs. No bilateral IUIs resulted from surgeries performed by an obstetric gynaecologist (Table 2).

**Table 2:** Descriptive statistics of IUIs and primary surgeon level of training

Level of training	No. of cases n = 12(%)	Total IUIs n = 19(%)	Unilateral IUIs n = 5(%)	Bilateral IUIs n = 14(%)
Medical officer	7 (58.3)	13 (68.4)	1(7.7)	12 (92.3)
Specialist surgeon	1 (8.3)	1(5.3)	1 (100)	0 (0)
Specialist OBGY	2 (16.7)	2 (10.5)	2 (100)	0 (0)
Specialist Public Health	2 (16.7)	3 (15.8)	1 (33.3)	2 (66.7)

Table 3 shows a summary of the individual case presentation, estimated time taken to make the diagnosis of injury, treatment administered at our centres, type of ureteral injury detected, the procedure performed, and post-repair complications.

**Table 3:** Summary of cases diagnosed and managed

Patient ID	Sex	Presentation	Time lag/h	ureteral injury detected at surgery	Procedure done	Complications	#
1^s^	F	Anuria -Anaemia -obstructive nephropathy	72-transfused	Lt Traction in a unilateral kidney	Release of traction suture + stenting	Reactionary haemorrhage	12
2^s^	M	Urine leakage	Intra-op	Rt. transection	Uretero-Ureterostomy and stenting	✔ Wound infection	21
3^ns^	F	Anuria, Urine peritonitis	48	Lt. transection Rt. ligation	Bil. Uretero-Neocystostomy (Lt. Boari-flap + Psoas Hitch) and stenting	✔ Wound infection	21
4^ns^	F	Anuria, Urine peritonitis	72	Bil. transection	Bil. Uretero-Neocystostomy + stenting	✔ Re-lap for leak ✔ Wound infection ✔ Ileus	32
5^ns^	F	Anuria	48	Bil. ligation	Bil. Uretero-Neocystostomy + stenting	✔ Ileus ✔ Wound infection	14
6^ns^	F	Anuria, Urine peritonitis	72	Lt. transection Rt. ligation	Bil. Uretero-Neocystostomy + stenting	✔ Wound dehiscence	21
7^ns^	F	On table suspicion	Intra-op	Lt traction	Release of a traction suture	✔ Bleeding ✔ Wound infection	21
8^ns^	F	On table	Intra-op	Bil. ligation	Bil. Uretero-Neocystostomy_stenting	✔ Wound infection	21
9^s^	F	Urine leakage	Intra-op	Lt. transection	Ureteroneocystostomy	None	14
10^ns^	F	Anuria Urine peritonitis	72	Right traction, left transection	Release of right traction stitch left ureteroneocystostomy + stenting	✔ Wound infection	14
11^ns^	F	Urine peritonitis Renal failure anaemia	72	Lt transection Rt ligation	Bil. Ureteroneocystostomy + JJ stenting	✔ Wound infection	21
12^s^	F	Urine peritonitis	Intra-op	Lt transection	Ureteroneocystostomy + JJ stenting	✔ Wound infection	14

Lt = left; Rt = right; Bil = bilateral; # = mean duration of hospital stay (days) = 18.7; s = specialist rank of primary surgeon (obstetric gynaecologist or general surgery specialists); ns = non-specialist rank of primary surgeon (medical officer, physician, public health specialist); re-lap = relaparotomy

Anuria, renal function derangement (obstructive uropathy), and urinary peritonitis were the common post-operative presentation while urine leakage during the surgery was the commonest sign for the intra-operative diagnosis. Five out of the seven cases of those who had bilateral injuries cases presented with both anuria and urinary peritonitis. Each of these cases had both ligation and transection injuries as depicted in Table 3. Seven out of the 12 cases of IUI were diagnosed post-surgery with 5 and 2 cases detected within 72 and 48 h respectively. The rest (five out of the 12 cases) were detected intraoperatively due to suspicion by the surgeon or detection of urine leakage.

Regarding the management of the ureteral injuries, all 19 ureteric injuries (seven out of the 12 cases being bilateral ureteric injuries) were repaired through open surgeries. Fourteen out of 19 injuries including all bilateral injuries occurred from hysterectomy surgery (63.2% from hysterectomy for uterine fibroids and 12.5% for post-partum haemorrhage). For the unilateral injuries, two resulted from hysterectomies for multiple uterine fibroids. All bilateral injuries and one unilateral injury occurred during surgery by non-specialist surgeons at the primary level. The left ureter was frequently injured in unilateral cases. The common injuries were transection injury, 9/19 (47.4%), and ligation, 7/19 (36.8%) of the ureters. Traction injury occurred in 3 out of 19 injuries. Most injuries occurred on the left distal ureter 11/19 (57.9%) as shown in Table 3.

For the definitive management of these cases, most of the patients had ureteroneocystostomy done with three having had the release of traction suture (Table 3). Wound infection was the most typical postoperative complication that occurred in seven of the 12 patients reviewed. One patient had a urine leakage that resulted in a prolonged hospital stay (32 days). Relaparotomy was carried out on one patient. The average duration of the hospital stay was 18.9 days.

## Discussion

Anatomically, the ureters are tubular structures with an approximate length of 25 cm extending from the renal pelvis to the urinary bladder trigone and located in the retroperitoneal space [9, 10]. The abdominal ureter initially lies on the anterior surface of the psoas muscle and descends postero-lateral crossing over the iliac vessels, with varied relationships to the pelvic brim. The ovarian vessels cross over the ureters anteriorly as they approach the pelvis. As the ureters enter the pelvis, the right crosses the external iliac artery and the left ureter crosses over the common iliac artery. They continue their course medial to the anterior division of the hypogastric artery and lateral to the peritoneum of the cul-de-sac into the midplane of the pelvis [9, 10]. The uterine arteries cross the ureters anteriorly before they tunnel into the cardinal ligaments to the uterus. This point is approximately 1.5 to 2.0 cm lateral to the internal cervical os and vaginal fornices before the ureters enter the trigone of the bladder [9, 10]. The relationship of the ureters with these vessels especially the distal third makes it vulnerable to damage during surgical procedures in the pelvis [8].

Ureteral injury may occur when suturing an extended incision aimed to control bleeding within the broad ligament or in performing a hypogastric artery ligation [10]. Common sites that the ureter is injured include the point where the ureters cross the pelvic brim, over the iliac arteries, within the cardinal ligament, and at the anterolateral fornix of the vagina [10]. Damage related to haemostatic sutures and dissection can be prevented by taking note that the renal artery, ovarian artery, common iliac artery, and aorta contribute to the blood supply to the ureter medially in the pelvic segment and laterally in the abdominal segment [9]. The peritoneum and interstitial/serosa layers should always be preserved during ureteral dissection. The outcome of management is worse in late diagnosis, coagulation and devascularization aetiologies, longer length damage, and in women [11].

This study reviewed IUI resulting from open surgeries managed by the urology units of two hospitals with expertise in open ureteric reconstructive surgery in that rural part of Ghana. Open abdominal hysterectomy was the most common surgery (10/12) in the elective setting due to uterine fibroid, uterine fibroid with infertility and menorrhagia, and in the emergency setting postpartum haemorrhage. This trend is similar to the report by Abboudi et al. (2013) and tertiary hospital reviews in urban Ghana [11] but differ from reported cases in Nigeria where mishaps during caesarean section led to 60% of the injuries [12–14].

Injury to the left ureter was the most common (57.9%) in our study. The variable course of the left ureter that brings it close to pelvic structures and the predominant right-handiness of surgeons have been considered to predispose to the frequent damage observed even though this is not conclusive [15].

The risk factors reported by primary surgeons that predispose to these complications were adhesions from previous pelvic surgeries and haemorrhage during dissection. These factors are well-published predictive factors for difficult hysterectomy as reported with recommended guidance during the procedure [12, 16]. Even though primary surgeries conducted by both specialists and non-specialists resulted in ureteric injuries, practical application of the discussed anatomy and the proposed protocols appear better followed by specialist surgeons.

Transection, ligation, devascularization, crushing, and perforation are the types of injuries that occur. Transection (9/19) and ligation (8/19) were the commonest injuries recorded in the study. This may be due to failure to identify the ureter during dissection or transection of the ligated uterine artery or blind transfixing leading to obstruction of the ureter as the injuries occurred in the pelvic part. This trend reveals the need for continuous instruction in the steps of hysterectomy procedures in rural settings. The Committee of the American Association for the Surgery of Trauma classified these injuries for better management into Grade I—hematoma, contusion or hematoma without devascularization, Grade II—laceration; < 50% transection, Grade III—laceration; 50% transection, Grade IV—laceration; complete transection with < 2 cm of devascularization and Grade V—laceration; avulsion with > 2 cm of devascularization [17]. In this study, transection and ligation injuries counted for the complications of urinary peritonitis, obstructive nephropathy, and anuria (60%). This is similar to other studies [12, 16]. Anuria as a pointer to a diagnosis of bilateral ureteric injury or injury to a single functioning system may be initially considered to be due to peri-operative hypovolaemia until late.

The majority of the missed cases were diagnosed 48–72 h post-operatively. In cases with delayed diagnosis, urine peritonitis, septicaemia, obstructive uropathy, fistula formation, and ureteric stricture may occur which poses a challenge to management and carry a poor prognosis after surgery with higher chances of patients developing renal hypertension, renal failure, fistula, urinoma formation, and recurrent ureteral stricture [11, 18]. The diagnosis in these cases was based on the presenting clinical symptoms, supported by blood urea nitrogen and creatinine elevation, neutrophilia, hydronephrosis, and free peritoneal fluid on ultrasound scan. These were confirmed by findings at exploration. The lack of advanced imaging and endourological investigation may potentially exclude minor injuries indicated in the Committee of the American Association for the Surgery of Trauma classification [13] being reported in our setting.

Patients with urinary peritonitis and obstructive nephropathy responded well to the intervention with only a single case of acute renal failure requiring dialysis. No long-term complications were noticed during the follow-up period.

In the present study, all the cases with bilateral injuries had the primary surgery performed by non-specialist medical practitioners. Thus there could be such cases undiagnosed in the rural setting as unilateral injuries potentially show equivocal symptoms or show negative findings during ultrasound imaging and so go unnoticed [12].

The outcomes of the patients with a deranged renal function who had re-laparotomy and those with normal renal function were similar.

Options of treatment for these ureteric injuries include percutaneous urinary diversion for injuries with obstruction in patients not fit for immediate exploration, the release of the stitch and double pig-tail stenting for traction injury without ischaemia, and immediate or delayed open surgical repair [2, 5, 6, 11, 16]. Laparoscopic and robot-assisted repairs have also been reported [19–21].

For distal ureteric injuries, aside endoscopic approach, options available include ureteroneocystostomy with psoas hitch, a Boari tubularized bladder flap, transureteroureterostomy, and in rare cases renal autotransplantation or ureteral substitution with a gastrointestinal segment [1]. In this study, the patients were managed by open surgical exploration with refluxing ureteroneocystostomy (14/19), Boari tubularised flap (1/19), and ureteroureterostomy for a mid-segment damage and suture release. All these repaired ureters were stented for a period. The minimum and maximum hospital stay were 14 days and 32 days respectively with an average of 18.7 days. All the patients had resolution of symptoms and normal renal function before discharge.

The common complication observed was wound infection that resolved with antibiotics in 10 out of 12 cases. In addition, we recorded 2 post-operative haemorrhages, 1 wound dehiscence, and the need for a second repair operation in one. There was no mortality.

## Limitation

This is a retrospective study with case selection criteria that may have resulted in the low incidence recorded. Our diagnosis of injuries was based on the evaluation of data on referred cases and did not capture cases that were primarily repaired without a referral. Conservatively managed cases or referred cases without clinical findings necessitating surgical exploration and repair were not included and could affect the numbers.

## Conclusion

Open hysterectomy (83.7%) was the most common surgery leading to iatrogenic ureteric injury in this study in a rural setting. Intraoperative recognition was mostly in trained specialist surgeons. Late presentation with more severe morbidity was found in non-specialist surgeons. Thus, improvement in training to allow intra-operative diagnosis should be encouraged in non-specialists to reduce morbidity and improve outcomes.

## Data Availability

The datasets used and/or analysed during the current study are available from the corresponding author on request.

## Abbreviation

IUI: Iatrogenic ureteric injury
